# The ENCODE Blacklist: Identification of Problematic Regions of the Genome

**DOI:** 10.1038/s41598-019-45839-z

**Published:** 2019-06-27

**Authors:** Haley M. Amemiya, Anshul Kundaje, Alan P. Boyle

**Affiliations:** 1 Graduate Program in Cellular and Molecular Biology, University of Michigan, Ann Arbor, MI USA; 2 Department of Computational Medicine and Bioinformatics, University of Michigan, Ann Arbor, MI USA; 3 Department of Genetics, Stanford School of Medicine, Stanford, CA USA; 4 Department of Human Genetics, University of Michigan, Ann Arbor, MI USA

**Keywords:** Genome informatics, Computational biology and bioinformatics

## Abstract

Functional genomics assays based on high-throughput sequencing greatly expand our ability to understand the genome. Here, we define the ENCODE blacklist- a comprehensive set of regions in the human, mouse, worm, and fly genomes that have anomalous, unstructured, or high signal in next-generation sequencing experiments independent of cell line or experiment. The removal of the ENCODE blacklist is an essential quality measure when analyzing functional genomics data.

## Introduction

The coupling of high-throughput technology with classic genomics assays enables us to study genome-wide architecture and regulation. Assays using high-throughput sequencing as a read-out of a genomic signal rely on an accurate genomic annotation and mapping. Inconsistencies in the underlying annotation exist at regions where assembly has been difficult. For instance, repetitive regions may be collapsed or under-represented in the reference sequence relative to the actual underlying genomic sequence. Resulting analysis of these regions can lead to inaccurate interpretation, as there may be significant enrichment of signal because of amplification of noise^1,2^. Problematic regions such as these have generally been ignored and unfiltered because they have not been found to affect the signal in the final analyses. However, in functional genomics assays such as chromatin immunoprecipitation followed by genome sequencing (ChIP-seq), accuracy in peak calling and downstream analyses is essential. Alignments in these problematic regions should be identified and filtered before application of any threshold, normalization, or peak calling as they can dramatically bias the results^2^.

The use of exclusive regions of “blacklists”, or regions where genome assembly results in erroneous signal, to remove signal-artifact regions in ChIP-seq experiments has been employed throughout the ENCODE project production phase^1,3,4^. The original ENCODE blacklist, termed the Duke Excluded Regions (DER), was manually curated on the *Homo sapiens* (human) genome assembly GRCh37 (hereafter referred to as hg19) to cover a large number of repeat elements in the genome, particularly rRNA, alpha satellites, and other simple repeats. This list was further updated, now referred to as ENCODE Data Analysis Center (DAC) blacklisted regions, to include regions of high signal that presumably represent unannotated repeats in the genome. The removal of these regions eliminated significant background noise that otherwise would have been thought to have been due to biological variation^2^. While this list was comprehensive, a significant amount of manual annotation was required to generate the final set of regions that would be laborious to apply to updated builds. The affected regions were broad, covering on average 45 kb with the largest being 1.4 Mb. Additionally, artifact regions are not human genome specific, and there was a need for identification of organism-specific regions.

To generate blacklists in an objective and systematic manner, we developed an automatic procedure to flag regions that appear to have artifact signal. Regions are flagged using uniform criteria applied across a large number of samples. All ENCODE, mouse ENCODE, and modENCODE input ChIP-seq samples (control data for ChIP-seq) were used for ENCODE (*Homo sapiens*: hg19 and the updated assembly GRCh38/hg38), mouse ENCODE (*Mus musculus:* mm9 and mm10), and modENCODE (*Caenorhabditis elegans:* ce10 and ce11, *Drosophila melanogaster:* dm3 and dm6) analyses, respectively. To identify regions for inspection, our method searches for regions that provide the signature of existing in multiple copies and are thus overrepresented in control “input” sequences. These “input” datasets were generated as controls for ChIP-seq experiments using randomly sheared DNA regions from non-immunoprecipitated chromatin. We examined all 1 kb windows with 100 bp overlap to identify such regions. Input samples are scored with input read depth and mappability, quantile normalized, and the median signal is selected (See methods). This defines a comprehensive and cell-type agnostic signal across the genome that is unaffected by high signal from a particular cell-line (eg. CNVs) or low signal due to differential processing of input data. Regions with read depths or multi-mapping read rates in the top 1% are considered likely artifacts (Supplemental Fig. 1). In all cases, the mitochondrial DNA and any reads mapping to these sequences are pre-filtered from analysis and are considered part of the blacklist.

**Figure 1:**
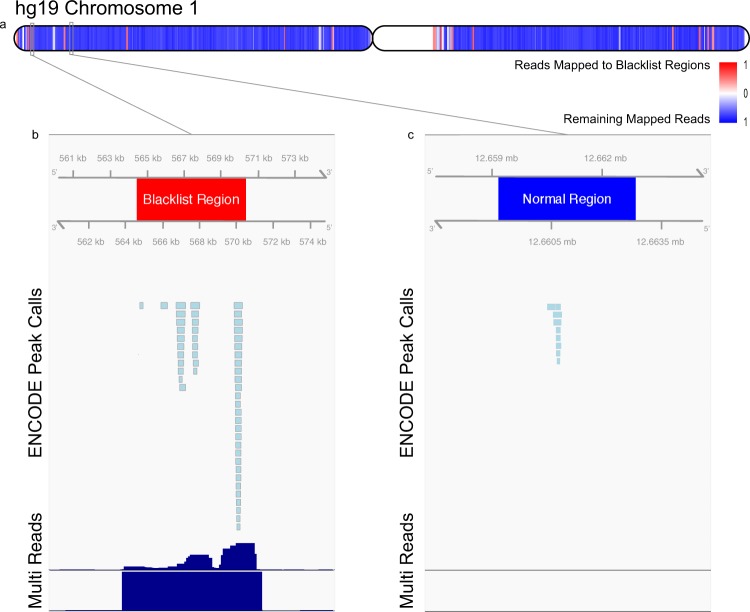
Blacklist regions are tightly distributed across the chromosome and sequester high read mapping signals. (**a**) Distribution of mapped reads along human chromosome 1 in hg19. (**b**) An example blacklisted region on chromosome 1. Displayed are pre-filtered ENCODE ChIP-seq peak calls, quantile normalized median read signal (Reads), and quantile normalized median multimapped read signal (Multi). Axes are scaled for illustrative purposes and signal values are truncated at approximately 10-fold enrichment. Signal in these regions are up to 6400× background levels. (**c**) An example “normal” ENCODE ChIP-seq peak region on chromosome 1 selected as a region containing ChIP-seq peaks.

**Figure 2:**
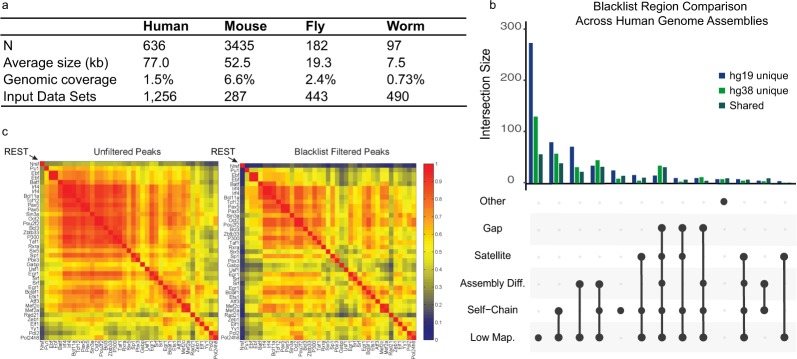
Blacklist regions account for a significant portion of ChIP-seq reads, are driven by artifacts in genome assemblies, and removal of these regions is essential to removing noise in genomics assays. (**a**) The number of blacklisted regions across species with their average size, genomic coverage, and input datasets excluding assembly gaps used for hg38, mm10, dm6, and ce11 respectively. (**b**) An UpSet plot displaying the breakdown of uniquely annotated regions in hg19 and hg38, and the shared regions between them. Low-mappability (Low-Map.) regions account for the majority of unique regions in both hg19 and hg38. (**c**) Applying the blacklist to ChIP-seq peaks results in an overall reduced correlation and, in the highlighted example, results in a more biologically meaningful interpretation of the data.

A blacklist was built for the human, mouse, worm, and fly genomes using all reads from input samples. In each organism, only a small portion of the genome was flagged as containing artifact sequence signal (Figs 1 and 2a). However, these regions were enriched for ChIP-seq reads in ChIP experiment for transcription factors (Fig. 1) and are particularly enriched for reads and peaks from lower quality experiments. In fact, ENCODE uses this as a quality control metric with some experiments having up to 87% of reads falling into blacklisted regions^5^. In Fig. 1 we show the distribution of all input reads mapped across chromosome 1, where reads mapping to blacklist regions are represented in red. The signal at blacklist regions are extremely high even though they account for a small fraction of the mappable chromosome (Fig. 1b). For example, this represented 582 million of 2.5 billion uniquely aligning reads mapping to blacklisted regions in the human ENCODE ChIP-seq data in hg19. These findings emphasize the extreme nature of these artifact regions and highlight the importance of filtering these regions to avoid incorrect biological conclusions.

We investigated the underlying characteristics of our automated hg19 blacklisted regions and their agreement with previously published lists^6^, which included our manually curated hg19 blacklist (DAC) (Supplemental Fig. 2). Though satellite repeats were used in the original ENCODE blacklists, they represent a small portion of the automated hg19 blacklist and are generally repeated in the genome annotation (Supplemental Fig. 2c). Additionally, they are not uniquely mapped with the algorithms used which prevents alignments to these regions. The automated hg19 and DAC blacklists detect the vast majority of regions flagged by a similar and complementary technique^6^ (Supplemental Fig. 2b). Of the regions unique to the automated hg19 blacklist, all cover gaps in the genome assembly (Supplemental Fig. 2c), lending evidence that these regions of the genome are incomplete in the hg19 assembly. Indeed, a large number of these regions were patched in the next iteration of hg19 or dropped in the GRCh38 assembly (Supplemental Fig. 2c). Almost all of the flagged regions also included nuclear mitochondrial DNA segments (NUMTs, Supplemental Fig. 2c), a criterion that was overlooked in the initial manual blacklists. There are many mitochondrial genomes in comparison to the singular nuclear genome, leading to a high read depth of NUMTs. Additionally, NUMT sequences are scattered throughout the genome, contributing to overrepresentation of NUMTs in the input sequence. For these reasons, it is critical to include these sequences in the blacklist. A majority of the regions that were flagged by the DAC blacklist but missed in the automated hg19 blacklist were defined by repeatmasker class annotations as Satellite repeats (Supplemental Fig. 2d). While many of these repeat regions contain anomalous signal, those that were excluded from the automated hg19 blacklist do not show high signal and are uniquely mapped in hg19. None of the regions unique to the DAC blacklist were patched or removed in the new assembly.

We next sought to characterize the differences between regions blacklisted from the automated pipeline in hg19 compared to the blacklist from the hg38 genome assembly. Generally, the same classes of regions are enriched in both assemblies. Many regions do not overlap due to assembly differences such as the expanded centromere and satellite sequences that are features of the hg38 assembly as well as fixed/new gaps that vary in both builds (Fig. 2b). A large portion of the differences occurs at low-mappability (Low Map.) annotations which represent short repetitive elements in the genome assembly that are poorly mappable and as a consequence do not map well between assemblies (Fig. 2b). Overall, these differences lead to the conclusion that the major differences between blacklists are due to underlying changes in the sequence assemblies. Consequently, this lends to the hypothesis that the driving factor behind artifact regions in the genome are due to issues in the assembly rather than other factors.

Finally, to demonstrate the artificial correlation created by these peak regions, we performed a correlation analysis of the ENCODE peak regions in the human genome using a blacklist-filtered set as well as an unfiltered set of peaks (Fig. 2c). In the unfiltered set of peaks, blacklist regions sequester a large portion of ChIP-seq reads, leading to an illusion of high correlation of these regions with others. After blacklist filtering, the correlation structure is more distinct. As a specific example, the correlation of REST (a known repressor) auto correlates with other TFs (most of them activating) without the filtering of the blacklist. The removal of the blacklist regions removes spurious correlations seen with REST has essentially disappeared as would be expected given the known biology. We highlight the clear case of removing the noise in REST correlation, but the same standard holds true for the remaining factors in Fig. 2c. The ENCODE blacklists have been used to filter all of the ChIP-seq data from the ENCODE project and improvements in data from the application of the blacklist to these data are a key evaluation metric used by the consortium. For a complete list of artifact effects on peaks from all ChIP experiments used in ENCODE, we have provided a reference to the ENCODE quality control metric spreadsheet^5^. Furthermore, another detailed analysis of the detrimental effects of not excluding these artifact regions has been previously described^2^. Biological validations of the most robust signal regions will likely result in testing of these artifact regions, potentially resulting in incorrect biological conclusions. Therefore, identification of these regions and subsequent filtering lead to more accurate and stable results across experiments.

The method implemented here requires a significant amount of input sequencing data from different sources in order to generate an accurate blacklist. For our analyses, we use all available ENCODE ChIP-seq input datasets to estimate the genomic regions that have these artifact properties, and the use of multiple cell-types is important to avoid blacklisting regions that are specific to a single cell-type or tissue. We also caution that the blacklists are specific to each genome assembly and a lift over from an old assembly is not meaningful or valid. Finally, those studying genes in unmappable regions of the genome will find their data filtered by the blacklist. These regions account for ~3% of human protein coding genes that have previously been shown to be unmappable using short-read technologies^7^.

We present a resource of genomic regions that should be identified and either filtered from study or analyzed independently for better understanding as to their potential regulatory function. It is important to note that we do not propose a single blacklist that can encapsulate error defined across all NGS based assays. The presented blacklist shows high concordance between chromatin-based filtering (DNase/ATAC-DAC blacklist) and ChIP-seq input based filtering. This is not surprising given that the input DNA for ChIP-seq has been shown to be a proxy for lightly digested open chromatin assays^8^. However, the same criteria cannot be applied for whole genome sequencing (WGS) filtering and RNA-seq filtering. WGS filtering does not result in poorer annotations if there are higher read depths in regions, and therefore this method would be counterproductive to genome assembly. In the case of RNA-seq, more cell-type specific corrections for copy number are appropriate as there is virtually no overlap of coding regions with existing blacklist regions. The method presented is employed by the ENCODE project, as well as many other established analysis pipelines, and allows for a noise filtering on DNase-seq, ATAC-seq, and ChIP-seq datasets to help improve the accuracy of studies using these data. The removal of blacklists differs from the typical removal of signals from duplicate reads since these regions are problematic across different cell types and individual experiments. Ultimately, the removal of blacklists should be integrated within genomic assay analysis pipelines that incorporate high-throughput sequencing in order to assess biologically relevant and true signals.

## Methods

### Selection of input datasets

All data were acquired from the ENCODE Data Coordination Center. Using a previously published perl script (https://github.com/Boyle-Lab/ENCODE-API-Apps)^9^, we queried the ENCODE DCC API for unfiltered bam files labeled “input” that were released for the correct genome assembly. In the case of humans, these bam files were merged based on ENCODE assigned donor accession numbers to collapse data by cell type or individual. This was performed using ‘samtools sort’ to first sort all samples, ‘samtools merge’ to merge, and finally ‘samtools index’ to generate a new index of the resulting bam files^10^.

### Generation of mappability data

The Umap tool was used to identify all positions on both strands of a target genome for which reads of a desired length starting at that position are uniquely mappable^11^.

### Building the blacklist

For each input dataset (or merged input for human) from ENCODE, the number of reads per mappable base and the number of multimapping reads per million reads is calculated for each bin of 1 kb with 100 bp overlap across all chromosomes. The values across bins are then quantile normalized and a standard value at the 50% quantile is selected to represent each bin. This threshold was selected to avoid high signal outliers from individual cell types (for example, from copy number variants) and to avoid low signal from failed or incorrectly labeled input datasets. The standard values across the genome are then flagged if they are in the top 0.1% of signal for either read depth or mappability. Neighboring regions are merged if they maintain a signal in the top 1% of all signal or if they have no signal due to no mappability in the genome and any flagged regions within 20 kb were combined. This generates contiguous regions of abnormal signal across the genome.

## Supplementary information

SUPPLEMENTARY INFO

## Data Availability

The blacklist software and called regions for multiple species are made available at https://github.com/Boyle-Lab/Blacklist/ and at the ENCODE DCC for human and mouse (https://www.encodeproject.org/annotations/ENCSR636HFF/).

## References

[1] ENCODE Project Consortium *et al*. An integrated encyclopedia of DNA elements in the human genome. *Nature* **489**, 57–74 (2012). 10.1038/nature11247 PMC3439153 22955616

[2] Carroll TS, Liang Z, Salama R, Stark R, de Santiago I (2014). Impact of artifact removal on ChIP quality metrics in ChIP-seq and ChIP-exo data. Front. Genet..

[3] Boyle AP (2014). Comparative analysis of regulatory information and circuits across distant species. Nature.

[4] Yue F (2014). A comparative encyclopedia of DNA elements in the mouse genome. Nature.

[5] https://docs.google.com/spreadsheets/d/1G4SkqUMiGcUlvR6homc7RW33nSOf4mS9QYJifsd4qo0/.

[6] Pickrell JK, Gaffney DJ, Gilad Y, Pritchard JK (2011). False positive peaks in ChIP-seq and other sequencing-based functional assays caused by unannotated high copy number regions. Bioinformatics.

[7] Li W, Freudenberg J (2014). Characterizing regions in the human genome unmappable by next-generation-sequencing at the read length of 1000 bases. Comput Biol Chem.

[8] Auerbach RK (2009). Mapping accessible chromatin regions using Sono-Seq. Proc Natl Acad Sci USA.

[9] Diehl AG, Boyle AP (2016). Deciphering ENCODE. Trends Genet.

[10] Li H (2009). The Sequence Alignment/Map format and SAMtools. Bioinformatics.

[11] Karimzadeh, M., Ernst, C., Kundaje, A. & Hoffman, M. M. Umap and Bismap: quantifying genome and methylome mappability. *Nucleic Acids Research*, gky677 (2018). 10.1093/nar/gky677 PMC6237805 30169659

