# Supplementary material for: The ENCODE Blacklist: Identification of Problematic Regions of the Genome
Source: Sci Rep. 2019 Jun 27;9:9354. doi: 10.1038/s41598-019-45839-z (PMC6597582; doi:10.1038/s41598-019-45839-z)
Supplement: Supplementary file 1 — SUPPLEMENTARY INFO [file 41598_2019_45839_MOESM1_ESM.pdf]

Supplemental Information for:

The ENCODE Blacklist: Identification of Problematic Regions of the Genome

Haley M. Amemiya, Anshul Kundaje, Alan P. Boyle

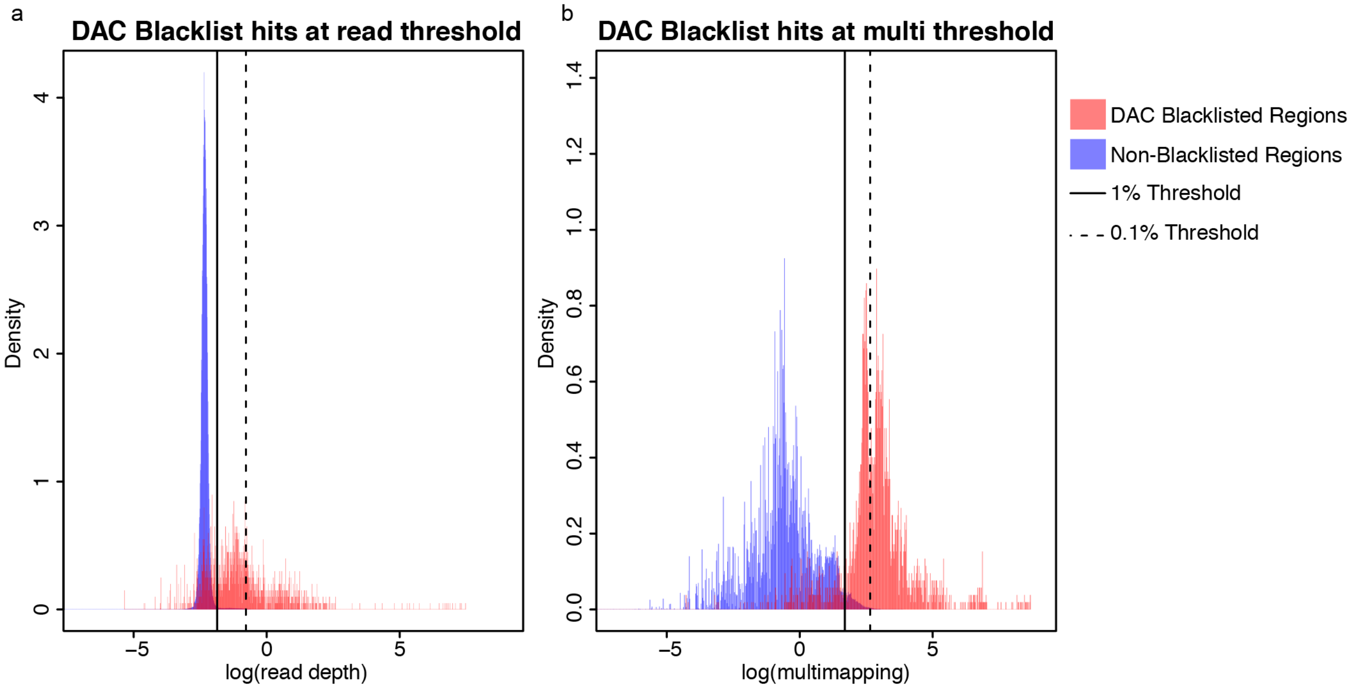

Supplemental Figure 1. Justification of thresholds for automated blacklist generation. The initial motivation behind the blacklist was to identify large artifact regions. These regions were envisioned as collapsed repeats in the genome that led to incredibly high numbers of reads. Early manual observations of these regions showed high levels of multimapping reads, high levels of reads, and multiple identical reads, and these manual observations generated what became the DAC blacklisted regions. Often these regions were at signal levels several orders of magnitude higher than the rest of the genome. As a result, in our automated method, we implemented a 1kb window with 100bp overlaps. In an attempt to not significantly overshoot the borders of these regions, this approach maintains a large enough region to identify the high signal in these often multi-kb regions. Here we have generated histograms of all 1kb windows from chromosome 1 and marked the 1% thresholds (black line) used to demonstrate the very long tail and conservative nature of this selection. Blue regions in this plot represent all 1kb windows from chromosome one not annotated by the manual blacklist and red regions represent all windows annotated by the DAC blacklist. Note that these histograms represent overall density in each class so that distinctions can be seen in the sets, but that the blue set represents 2,488,826 windows while the red set represents only 1,671. There is a very clear delineation between the 1kb windows manually identified as artifacts from the rest of the genome, and this transition occurs at the 1% mark. Therefore, this threshold was selected as being optimal for automated genome-wide identification of blacklist regions.

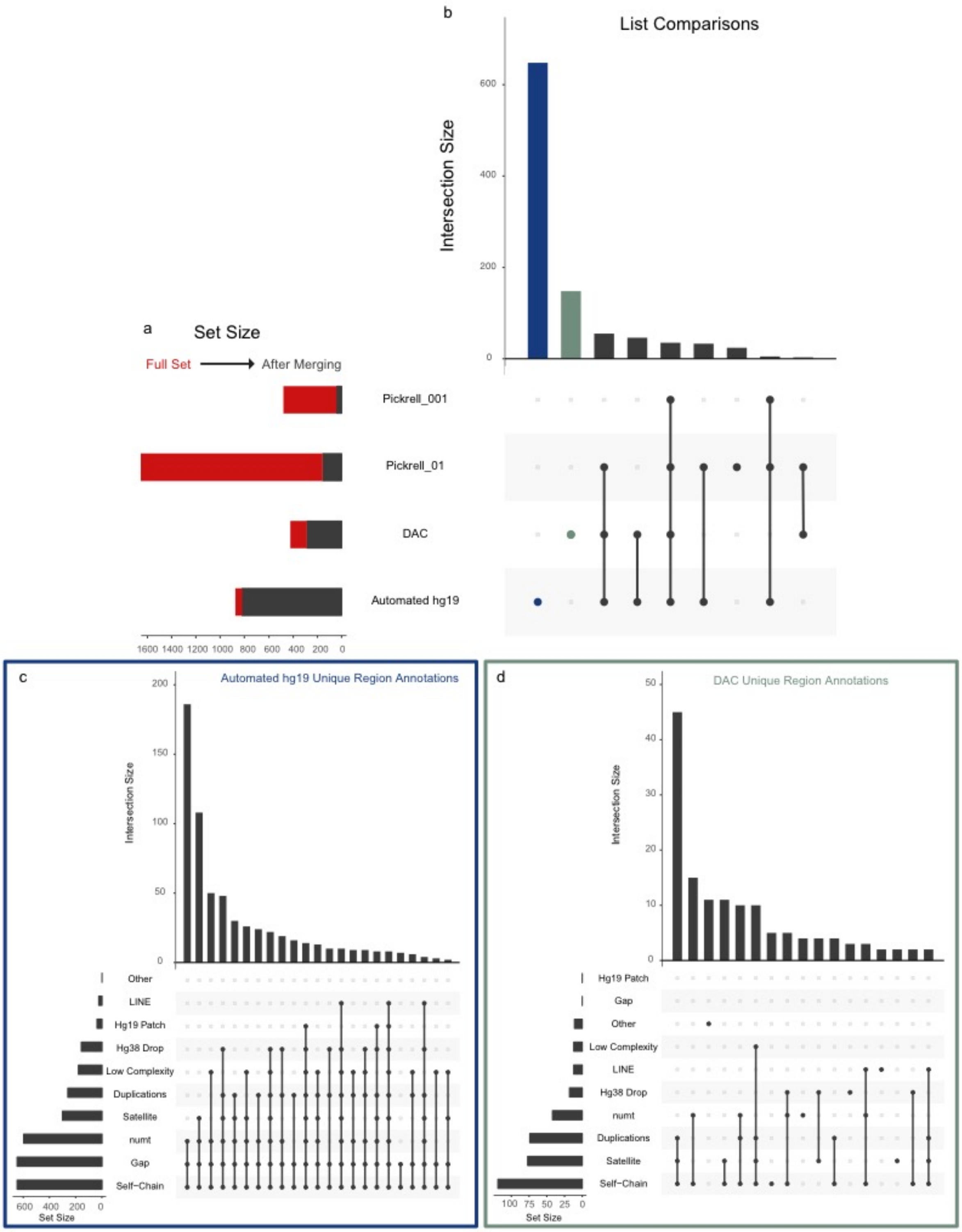

Supplemental Figure 2. Comparison across different “blacklists”. In order to better understand the types of regions being annotated, we studied the similarities and differences across our automated and manual blacklists in hg19 as well as an analysis done by Pickrell et al. to identify high-signal sites. a) In order to compare across disparate genomic intervals, we first merged lists of regions. Following merging, many of the lists became shorter due to many small regions overlapping larger annotations. b) An UpSet plot displays the number of unique regions when comparison across the sets. Notably, both the DAC and automated hg19 lists contained the most unique regions which we explored further. c) The automated hg19 unique regions consist of assembly changes and gaps, as well as a large number of nuclear mitochondrial DNA segments. These indicate regions that were problematic in the assembly and were changed in the more updated build of the genome. Furthermore, nuclear copies of mtDNA (numts) were not considered in the initial manual annotation and because of their

duplicative nature in the genome are likely to also have high signal. d) The unique regions in the DAC manual blacklist are primarily annotated as satellite repeats. While these regions are repetitive areas, they are mappable in the genome and do not display an aberrant signal. These were likely included from the original DER manual list that was primarily based on satellite annotations and not aberrant signal.
